# mtDNA haplogroup J Modulates telomere length and Nitric Oxide production

**DOI:** 10.1186/1471-2474-12-283

**Published:** 2011-12-15

**Authors:** Mercedes Fernández-Moreno, María Tamayo, Angel Soto-Hermida, Alejandro Mosquera, Natividad Oreiro, Carlos Fernández-López, José Luis Fernández, Ignacio Rego-Pérez, Francisco J Blanco

**Affiliations:** 1 INIBIC-Complejo Hospitalario Universitario A Coruña (CHUAC). Rheumatology Division, As Xubias 84, 15006-A Coruña, Spain; 2 INIBIC-Complejo Hospitalario Universitario A Coruña (CHUAC). Genetics Unit, As Xubias 84, 15006-A Coruña, Spain; 3 Proteo-Red/ISCIII. Madrid. Spain; 4 CIBER-BBN-ISCIII. Madrid. Spain

**Keywords:** mitochondria, osteoarthritis, cartilage, arthritis, nitric oxide, telomere

## Abstract

**Background:**

Oxidative stress due to the overproduction of nitric oxide (NO) and other oxygen reactive species (ROS), play a main role in the initiation and progression of the OA disease and leads to the degeneration of mitochondria. Therefore, the goal of this work is to describe the difference in telomere length of peripheral blood leukocytes (PBLs) and Nitric Oxide (NO) production between mitochondrial DNA (mtDNA) haplogroup J and non-J carriers, as indirect approaches of oxidative stress.

**Methods:**

The telomere length of PBL was analyzed in DNA samples from 166 healthy controls (114 J and 52 non-J) and 79 OA patients (41 J and 38 non-J) by means of a validated qPCR method. The NO production was assessed in 7 carriers of the haplogroup J and 27 non-J carriers, by means of the colorimetric reaction of the Griess reagent in supernatants of cultured chondrocytes. Inducible nitric oxide synthase (iNOS) mRNA from these samples was analyzed by qPCR. Appropiated statistical analyses were performed

**Results:**

Carriers of the haplogroup J showed a significantly longer telomere length of PBLs than non-J carriers, regardless of age, gender and diagnosis (p = 0.025). Cultured chondrocytes carrying the mtDNA haplogroup J also showed a lower NO production than non-J carriers (p = 0.043). No significant correlations between age and telomore length of PBLs were detected neither for carriers of the haplogroup J nor for non-J carriers. A strong positive correlation between NO production and iNOS expression was also observed (correlation coefficient = 0.791, p < 0.001).

**Conclusion:**

The protective effect of the mtDNA haplogroup J in the OA disease arise from a lower oxidative stress in carriers of this haplogroup, since this haplogroup is related to lower NO production and hence longer telomere length of PBLs too.

## Background

Osteoarthritis (OA), the most common form of joint disease and cause of musculoskeletal disability in elderly people, is a disease affecting articular cartilage, bone and soft tissue leading to joint destruction and severe impairment of mobility [1]. It is also the main cause of work incapacity and one of the most common reasons for visiting primary physicians. The metabolic and structural changes that take place in the articular cartilage, including the reactive oxygen and nitrogen species (RONS), are thought to play a main role in the initiation and progression of this disease.

A growing body of evidence suggests that oxidative damage, due to the overproduction of nitric oxide (NO) and other reactive oxygen species (ROS), may be involved in the pathogenesis of OA [2,3]. The increased levels of these ROS have been correlated to increased levels of inflammatory cytokines, such as interleukin-1 (IL-1), which is implicated in the degeneration of cartilage due to its induction of proteoglycan loss and matrix degradation [4]. Both IL-1 and mechanical loading of cartilage increase the production of NO by upregulating the nitric oxide synthase 2 [5]; and most of the destructive effects of NO in articular cartilage are related to the ability of NO to combine with superoxide anions (O_2_^-^) to generate peroxynitrite (ONOO^-^) [6,7].

RONS can have multiple effects on chondrocytes, but are associated with oxidative damage to DNA, proteins and lipids, resulting in a loss of extracellular matrix and cell death [2,8,9]. However, the most direct effect of NO is the inhibition of adenosine triphosphate (ATP) production, by competing with oxygen to bind to cytochrome oxidase on the mitochondria, thereby inhibiting the electron transport chain and the generation of ATP [10,11]. This latter aspect would strengthen the role of the mitochondria in the OA disease, as previously described [6,12-15].

The mtDNA haplogroups have been associated not only with several multifactorial diseases [16-18] and ageing [19,20], but also with OA; people carrying the mtDNA haplogroup J show lower prevalence and severity of knee and hip OA [14,21]. Besides, they modulate the serum levels of some collagen type-II molecular biomarkers [22] as well as some proteolytic enzymes, such as metalloproteinases [23]. The proposed mechanism relies on the different metabolic characteristics of these haplogroups, reflected by the performance of the mitochondrial oxidative phosphorylation system (OXPHOS) of each haplogroup [16,24], as well as the lower oxygen consumption and lower oxidative damage in carriers of the mtDNA haplogroup J [25].

Telomeres are capping structures at chromosome ends that prevent the recognition of natural chromosome ends as DNA double strand breaks. After several cell doublings, excessive telomere shortening triggers a checkpoint, leading to apoptosis. However, it has also been reported that oxygen free radicals (O_2_^- ^and ONOO^-^) directly injure the guanine repeats in the telomere DNA, indicating that oxidative stress directly leads to telomere erosion, regardless of cell division [26,27].

Taking into account that mtDNA haplogroup J is less prone to suffer oxidative stress than the rest of mitochondrial haplogroups, as previously proposed [24,28,29], the aim of this study was to evaluate if this mtDNA haplogroup is correlated with both the telomere length of peripheral blood leukocytes (PBL) and the NO production, two oxidative stress-related features. In order to demonstrate this, we measured the telomere length of peripheral blood leukocytes (PBLs) in a large cohort of samples (OA patients and healthy controls), and assessed the NO production and inducible Nitric oxide synthase (iNOS) expression in cultured chondrocytes.

## Methods

### Samples obtention

For telomere length, DNA from healthy (hip OA-free) control population (n = 115; mean age = 43.13 ± 12.47 years; range: 19-68) was provided by Banco Nacional de ADN (University of Salamanca, Spain), and also by Hospital Universitario A Coruña ((n = 79 OA patients; mean age = 69.46 ± 8.97 years; range: 42-95) and (n = 51 healthy controls; mean age = 71 ± 14.48; range: 42-93)). Total of 166 healthy controls (89 females and 77 males), 114 had the haplogroup J and 52 were non-J carriers; of the 79 OA patients (54 females and 25 males), 41 had the haplogroup J and 38 were non-J carriers. Those healthy controls from Banco Nacional de ADN consisted of a population sample on which only those individuals who did not suffer from symptomatic hip OA neither had been diagnosed of hip OA at the moment of the study were selected. The cohort from A Coruña include hip OA patients with different radiographic K-L grades, and controls free of hip OA as assessed by anamnesis, a clinical examination and radiographic studies.

For NO production and iNOS expression, the cartilage samples were also obtained from Hospital Universitario A Coruña: OA cartilage was obtained from the femoral heads of 24 OA patients (12 females and 12 males) who underwent joint replacement surgery (mean age = 73.61 ± 8.39 years; range: 57-90), and normal human cartilage was obtained from autopsy from 10 cadavers (6 females and 4 males) who had no history of joint disease and who had macroscopically normal cartilage, as well as from patients who suffered hip fracture and underwent hip replacement surgery (mean age = 63.90 ± 14.26 years; range: 45-83). Of the 24 OA patients, 4 have the haplogroup J and 20 are non-J carriers; of the 10 healthy controls, 3 have the haplogroup J and 7 are non-J carriers. Informed consent was obtained from all participants, and this study was approved by ethics committee of Galicia (reference number 2008/141).

### DNA isolation

DNA from samples obtained in our facilities was extracted using the Magtration System12CG (Precision System Science Co., Ltd., Matsudo, Chiba, Japan) using Magtration-MagaZorb DNA Common Kit-200 N reagents, and then quantified using the NanoDrop ND-1000 Spectrophotometer (NanoDrop Technologies, Wilmington, Delaware, USA). Coded DNA samples were processed by personnel blinded to the status of the subjects. The isolated DNA was used to perform both the assignment of the mtDNA haplogroups and the telomere quantification.

### Assignment of mtDNA haplogroups

The samples obtained for the present study were haplogroup-typed using a previously described assay [14].

### Telomere quantification

The average telomere length of PBL was measured with a validated quantitative (Q-PCR)-based assay using a LightCycler 480 thermalcycler (Roche Diagnostics^®^, Laval, Quebec, Canada) in a 96-well format, as described in Tamayo *et al *[30]. This method measures the average ratio of telomere repeat copy number to a single gene (36B4) copy number (T/S ratio) in each sample.

### Chondrocytes culture and NO production

The chondrocytes culture was performed as described in Maneiro *et al *[12]. Briefly, the dissected cartilage was incubated at 37°C with trypsin for 10 minutes. After removing trypsin solution, the cartilage slices were treated with type IV collagenase (2 mg/mL; SIGMA, St. Louis, MO) for 12-16 hours. Human chondrocytes were recovered and plated in Dulbecco's modified Eagle's medium (Life technologies, Paisley, UK) supplemented with 100 units/ml penicillim, 100 μg/mL streptomycin and 10% fetal bovine serum (FBS) (Life technologies). Chondrocytes were incubated at 37°C in a humidified gas mixture containing 5% CO_2_.

When human chondrocytes reached the confluency, a total of 5 × 10^4 ^cells were collected by trypsinization and plated in 96-well plates with Dulbecco's modified Eagle's medium supplemented with 100 units/ml penicillin, 100 μg/mL streptomycin and 10% FBS for 24 hours. In order to measure the NO production, the FBS of the medium was changed to a concentration of 0,5% for 48 hours, after which the assessment of the NO production was carried out. The measure was performed by an indirect method in supernatants of each sample by duplicate by means of the colorimetric reaction of the Griess reagent (Enzo Life Sciences, Farmingdale, New York, USA), using a standard curve with NaNO_2_. The absorbencies emitted were captured in a spectrophotometer (*Labsystems Multiskan^® ^Plus*).

### iNOS expression

RNA was isolated from the above cultured cells (5 × 10^5^) following the Trizol (Invitrogen^®^, Life Technologies, Paisley, UK)-based method. Isolated and DNase-treated RNA (1000 ng) was retrotranscribed to cDNA using the Transcriptor First Strand cDNA synthesis Kit from Roche Diagnostics^® ^following the manufacturer's recommendations.

The real-time PCR was performed using a LightCycler 480 thermalcycler in a 96-well format. Duplicate cDNA samples were amplified in parallel in a final volume of 20 μL, that included 5 μL cDNA, 10 μL of LightCycler^® ^480 Sybr Green I Master (Roche Diagnostics^®^), 0.25 μL Uracil-DNA glycosylase (UDG) to prevent carry-over contaminations (Roche Diagnostics) and 0.3 μM of specific primers for iNOS (forward: 5'-gctgccaagctgaaattga-3'; reverse: 5'-gatagcgcttctggctcttg-3') and the reference gene HPRT (forward: 5'-tgaccttgatttattttgcatacc-3'; reverse: 5'-cgagcaagacgttcagtcct-3'). After two previous steps of 40°C for 10 minutes, to activate the UDG, and 95°C for 10 minutes to activate the Taq polymerase, the mixture was amplified as follows: 55 cycles at 95°C for 10 seconds, 60°C for 15 seconds and 72°C for 10 seconds; with a final extension of 72°C for 1 minute. The specificity of all reactions was determined by melting point curve analysis using one cycle at 95°C for 5 seconds and 65°C for 1 minute followed by a heating up step to 97°C in a continuous way of fluorescence acquisition.

### Statistical analyses

Statistical analyses were performed using SPSS software, release 17 (Chicago, USA) and Relative Expression Software Tool (REST), release 1.9.9 [31]. A non-parametric study was performed, utilizing the Mann-Whitney *U*-test, to compare the telomere length of PBL between OA patients and healthy controls, as well as between haplogroup J carriers and non-J carriers. The same statistical approach was used to compare the NO production and iNOS expression between haplogroup J and non-J carriers.

Following the above approach, an analysis of covariance (ANCOVA) was used to evaluate the effects of haplogroup J on telomere length, adjusting for the confounder effects of diagnosis, gender and covariate age. ANCOVA assumptions were tested in each case and no violations were found. Before the multivariate analysis, a distribution analysis using the Kolmogorov-Smirnov test showed that the telomere length data were normally distributed. Additionally, we also performed a Spearman's correlation test and subsequent scatterplots to analyze possible correlations between age and telomere length in both J and non-J carriers.

Finally, the data from the real time PCR experiments were analyzed using REST software, which uses bootstrap randomization techniques to determine whether an observed up or down-regulation in samples is significant after normalization to housekeepers. To test the correlation between the iNOS expression and the NO production, a Spearman's correlation analysis was performed.

## Results

### Telomere length

The non-parametric analysis of the telomere length of PBL between OA patients and healthy controls showed that OA patients had a shorter telomere length than healthy controls (0.986 vs 1.008 respectively), however this difference did not reach the statistical significance between both groups (Table 1). Interestingly, the mean telomere length for all the mtDNA haplogroups showed that carriers of the haplogroup J had a significantly longer length than non-J carriers (1.039 vs 0.933 respectively) (p = 0.003) (Table 1).

**Table 1:** Mean telomere length in OA patients and healthy controls and in carriers of the mtDNA haplogroup J

	n	Mean age ± SD	T/S ratio ± SD	p*
OA patients	79	69.46 ± 8.97	0.986 ± 0.261	0.684
Healthy controls	166	51.92 ± 18.45	1.008 ± 0.287	
Haplogroup J	155	52.97 ± 17.60	1.039 ± 0.287	0.003**
Non-J	90	65.22 ± 15.98	0.933 ± 0.254	

(*) Mann-Whitney non-parametric *U*-test

(**) indicates statistical significance (p ≤ 0.05)

SD = Standard deviation

**Figure 1:**
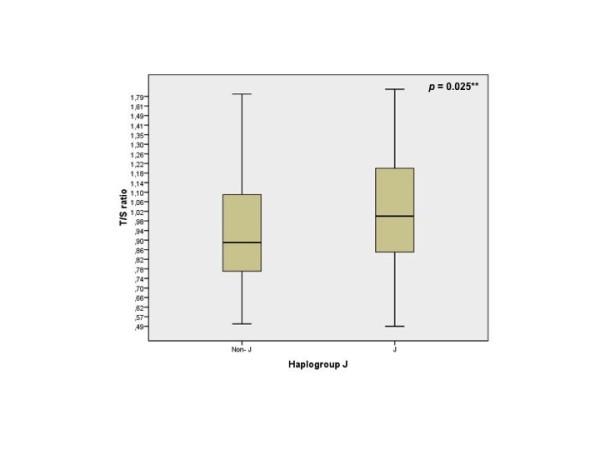
**Multiple regression analysis**. Telomere length between haplogroup J (n = 155) and non-J carriers (n = 90). (**: multiple regression analysis).

We then performed a multiple regression analysis to assess the effects of the mtDNA haplogroup J and other variables such as diagnosis, gender and age on the telomere length of PBL. The results obtained also reflected that carriers of the mtDNA haplogroup J showed a significantly longer telomere length than non-J carriers (p = 0.025) (Figure 1). The rest of variables tested such as age, gender and diagnosis did not show any influence on the telomere length of PBLs.

**Figure 2:**
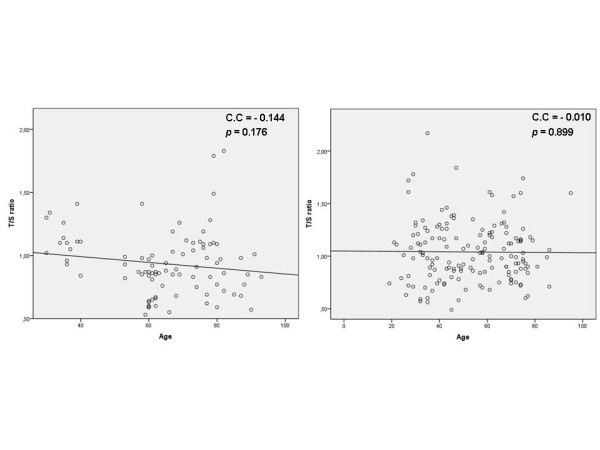
**Spearman's correlation test and scatterplots representing the correlation analysis between age and telomere length of PBLs**. **a) **non-J carriers and **b) **haplogroup J carriers. (C.C: correlation coefficient from Spearman's correlation test).

Despite age was not associated with telomere length in this study, we also performed a Spearman's correlation test and subsequent scatterplots for both J and non-J carriers. The results obtained did not show correlation between age and telomere length of PBLs neither in carriers of the haplogroup J neither in non-J carriers, strengthening the results obtained in the multiple regression analysis (Figure 2a and 2b).

### NO production and iNOS expression

**Figure 3:**
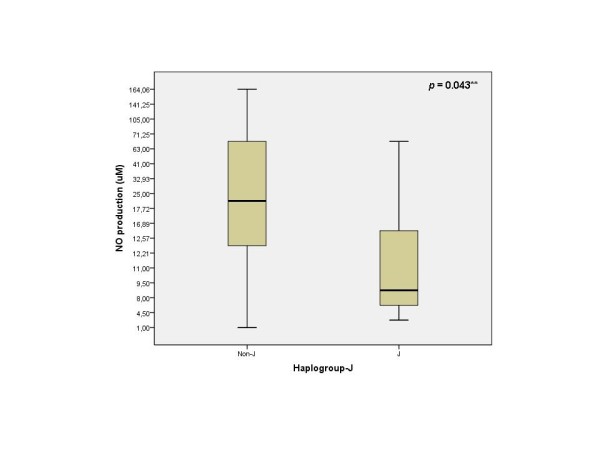
**Mean values of NO**. NO production between haplogroup J (n = 7) and non-J carriers (n = 27). (**: Mann-Whitney U test).

**Figure 4:**
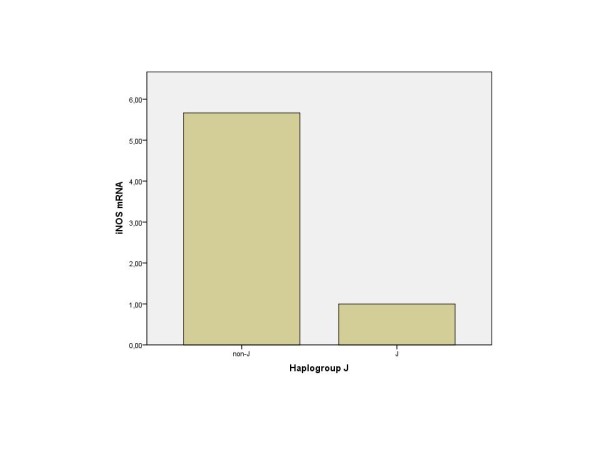
**Relative values of iNOS**. mRNA levels between haplogroup J (n = 7) and non-J (n = 27) carriers.

**Figure 5:**
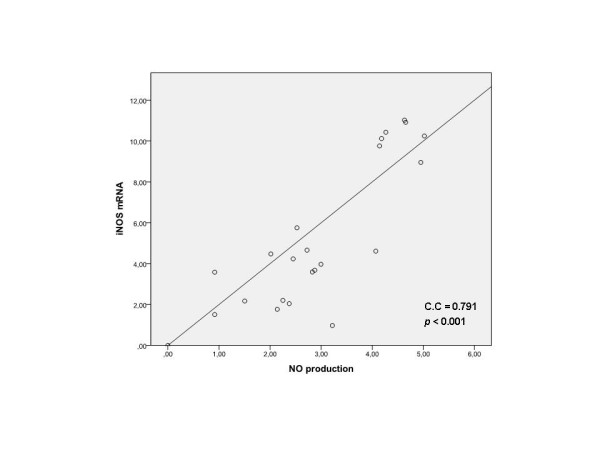
**Spearman's correlation test and scatterplots representing the correlation between iNOS and NO**. The correlation analysis is expressed as normalized iNOS mRNA values versus the NO production from cultured chondrocytes. (C.C: correlation coefficient).

The results obtained showed that mean NO production was significantly lower in haplogroup J carriers when compared with non-J carriers (17.75 vs 47.30 respectively; p = 0.043) (Figure 3). The expression of iNOS in carriers of haplogroup J was (not significantly) lower than in non-J carriers (Figure 4). In addition, a strong positive correlation between the NO production and the expression of iNOS was also demonstrated (correlation coefficient 0.791, p-value < 0.001) (Figure 5).

## Discussion

To our knowledge, this is the first study to correlate the telomere length with the mtDNA haplogroups, showing that carriers of the mtDNA haplogroup J have a longer telomere length of PBLs than non-J carriers. The results obtained also showed that cultured chondrocytes that carry the mtDNA haplogroup J show lower NO production and lower levels of iNOS than non-J carriers.

There are several studies that analyzed the telomerase activity in OA that showed that the presence of oxidative stress induces telomere genomic instability, replicative senescence and dysfunction of chondrocytes in OA cartilage [27]. On the contrary, other study proposes that the role of telomerase in the OA pathogenesis is still uncertain [32]. A recent work reported that telomere length of PBLs seemed not to be influenced by local OA pathology, confirming our present results; nevertheless, other rheumatologic diseases with higher and systemic inflammatory component, such as rheumatoid arthritis (RA), psoriatic arthritis (PA) and ankylosing spondilytis (AS) affected telomere length of PBLs, so that these patients showed longer telomere length than healthy controls [30]. However, the telomere length in OA chondrocytes is significantly shorter than in healthy aged chondrocytes, which may imply a local advance senescence that could contribute to the pathogenesis or progression of the OA disease [33].

It has been previously described that oxidative stress leads to telomere erosion, regardless of cell division, and that IL-1, one of the main pro-inflammatory cytokines involved in the OA process, stimulates NO production leading to the formation of ONOO^-^, which targets guanine repeats in DNA telomeres [26,27,34]. NO has been previously described to be increased in OA chondrocytes [35-37], and our results confirmed these findings (data not shown). In this sense, a work carried out by our group showed that carriers of the mtDNA haplogroup J have lower serum levels of Coll2-1NO_2 _than carriers of the haplogroup H [22] and, because the effect of NO on chondrocyte survival has been shown to be mediated by its effect on the mitochondrial respiratory chain [11], and Coll2-1NO_2 _is an indicator of oxidative stress status of the chondrocyte [38], these findings suggest that chondrocytes carrying the haplogroup J may have less oxidative stress. This latter conclusion is strengthen in the present study, since those carriers of the mtDNA haplogroup J show not only longer telomere length of PBLs, but also lower NO production and lower iNOS expression in cultured chondrocytes. However, we must point out that the telomere length was measured in PBLs and the NO production in cultured chondrocytes, therefore a direct correlation cannot be determined, but it is possible to speculate it since both cell types have in common the haplogroup J.

The proposed explanation would rely on the reduced coupling efficiency of the OXPHOS system of the mtDNA haplogroup J. This would reduce maximal ATP production and keep the mitochondrial electron transport chain more oxidized, thereby reducing ROS production and apoptosis [29]. With this scenario, the lower the O_2_^- ^generated, the lower the ONOO^- ^produced, resulting in a decreased oxidative damage by RONS and, consequently, the longer the telomere length. Besides, it is well known that mitochondria is involved in the NO production through the reduction of nitrite by cytochrome c oxidase, and regulated by oxygen on multiple levels [39,40]; since mtDNA haplogroup J show a lower oxygen consumption [25], we speculate that this could be one of the reasons why carriers of this haplogroup show lower NO production and hence longer telomere length, indicating a decreased oxidative damage. Because of this, the mtDNA haplogroup J not only protects from the development of both knee and hip OA [14,21], but has also been associated with increased longevity in different independent studies [19,20,41,42].

Despite the strong statistical correlation between the iNOS mRNA expression and the NO production, a higher variability in the results was detected. This could be due to i) the existence of different phenotypes of the OA disease and, among the possible causes to explain this, the mtDNA haplogroups could be one of them; or ii) the small sample size regarding to haplogroup J carriers. On the contrary, this variability was not detected when analyzed the telomere length of PBL, and a consistent statistical association between the mtDNA haplogroup J and the telomere length was detected, clearly indicating that cells carrying this haplogroup suffer less oxidative stress. However, no differences in the telomere length of PBLs were detected between OA patients and healthy controls, as described earlier [30].

## Conclusion

In summary, this study shows that carriers of the mtDNA haplogroup J show a significantly higher telomere length of PBL than non-J carriers, as well as a decreased NO production and lower iNOS mRNA levels in cultured chondrocytes, indicating that this haplogroup, which is clearly biochemically different from those of other population-specific mtDNA lineages [43], is associated with reduced oxidative stress. However, since these two features were analyzed in two different cell types, a correlation between the lower NO production and the longer telomere length cannot be directly determined but it is possible to speculate it because both cell types have in common the haplogroup J.

## Abbreviations

mtDNA: Mitochondrial deoxyribonucleic acid; PBL: Peripheral blood leukocytes; NO: Nitric oxide; qPCR: Quantitative polymerase chain reaction; ANCOVA: Analysis of covariance; iNOS: Inducible nitric oxide synthase; REST: Relative expression software tool; OA: Osteoarthritis; RONS: Reactive oxygen and nitrogen species; ROS: Reactive oxygen species; IL-1: Interleukin-1; O_2_^-^: Ion superoxide; ONOO^-^: Peroxynitrite; DNA: deoxyribonucleic acid; ATP: Adenosine triphosphate; OXHPOS: Oxidative phosphorylation system; FBS: Fetal bovine serum; cDNA: complementary deoxyribonucleic acid; UDG: Uracil-DNA glycosylase; RA: Rheumatoid arthritis; PA: Psoriatic arthritis; AS: Ankylosing Spondilytis; Coll2-1NO_2_: Nitrated form of the denaturation epitope of the triple helical domain of the collagen type II; SPSS: Statistical package for the social sciences.

## Competing interests

The authors declare that they have no competing interests.

## Authors' contributions

MFM carried out the experimental procedures of the mitochondrial haplogroups identification. She performed the statistical analysis and helped to draft the manuscript. MT and AM performed telomere quantification experiments. ASH carried out DNA isolation, quantification of Nitric Oxide production and iNOS expression. NO and CFL helped in collecting of samples and data clinic. JLF participated in study design and interpretation of data. IR was involved in the assignment of mtDNA haplogroups and analysed the data. He also participated in study design, interpretation of data and drafted the manuscript. FJB conceived and coordinated the project and drafted the manuscript. All authors read and approved the final manuscript.

## Pre-publication history

The pre-publication history for this paper can be accessed here:

http://www.biomedcentral.com/1471-2474/12/283/prepub
